# Using Patient-Reported Outcome Measures for Quality Improvement in Clinical Genetics: an Exploratory Study

**DOI:** 10.1007/s10897-017-0079-6

**Published:** 2017-03-09

**Authors:** A. Costal Tirado, A. M. McDermott, C. Thomas, D. Ferrick, J. Harris, A. Edwards, Marion McAllister

**Affiliations:** 1 Centre for Medical Education, School of Medicine, Cardiff University, Cardiff, UK; 2 University of Barcelona, Barcelona, Spain; 3 Cardiff Business School, Cardiff University, Cardiff, UK; 4 Cardiff & Vale University Health Board, Cardiff, UK

**Keywords:** Patient-reported outcome measures (PROMs), Quality improvement, Clinical genetics, Exploratory

## Abstract

International advocacy of patient-centred healthcare delivery has led to emphasis on the (re)design and evaluation of healthcare processes and outcomes from a patient perspective. Patient-reported outcome measures (PROMs) have significant potential to inform such attempts. However there is limited understanding of the processes by which this can be achieved. This exploratory study followed attempts to utilise two different PROMs measures to support service quality improvement in clinical genetics. PROMs used were the Genetic Counseling Outcome Scale (GCOS-24), a well-validated clinical genetics-specific PROM and Euroqol (EQ-5D), a generic PROM favoured by the UK National Institute for Health and Excellence (NICE). Both of these PROMs enable pre/post intervention comparison. A service audit tool was also used, premised on a patient-reported experience measure. In addition, the study draws on interviews with clinical staff to identify challenges associated with the use of PROMs (response rate, data collection, analysis). Benefits are also explored and include the provision of insight into patients’ needs; complementing clinical judgement; identification of needs being met, evidencing the benefit of services provided; prompting consideration of areas requiring attention; and encouraging professional development.

**Electronic supplementary material:**

The online version of this article (doi:10.1007/s10897-017-0079-6) contains supplementary material, which is available to authorized users.

## Introduction

Healthcare quality improvement refers to “designing and redesigning work processes and systems that deliver healthcare with better outcomes and lower cost” (Ham et al. 2016 p3). In the UK – and more broadly - there are two approaches to this: the centralised approach, emphasising regulation and inspection of providers, and the devolved approach in which healthcare quality improvement initiatives are led by local clinicians. Both approaches can incorporate use of patient-reported outcomes (Darzi 2008; Dawson et al. 2010; Leatherman and Sunderland 2008), enabling alignment with an international shift towards emphasising patient-centred healthcare delivery. For example, in 2008, the World Health Organization (WHO) reported that healthcare services should be more patient-centred (WHO 2008). The WHO claims that patients want more from healthcare than simply medical interventions, arguing that “People-centeredness is not a luxury, it is a necessity” (WHO 2008, p. 16). In the UK context of this study, the Department of Health (DH) published guidance in 2010, which mandated that the UK National Health Service (NHS) become more patient-centred. This guidance highlighted the importance of sharing decision-making between patients and professionals, with evaluation of patients’ perspectives important in this process (DH 2010). One way of assessing patients’ perspectives is using patient-reported outcome measures (PROMs). This is evident internationally, including in the USA, as exemplified by the National Institute of Health-funded Patient-Reported Outcome Measures Information Systems initiative (Ader 2007).

PROMs are short questionnaires completed by patients for assessment of health and health-related quality of life outcomes. PROMs were traditionally used in the context of clinical trials, but are increasingly being used to evaluate routine healthcare (Appleby and Devlin 2004; Dawson et al. 2010). PROMs data are collected directly from patients, without clinician interpretation, and paying attention to PROMs data is considered important for delivery of patient-centred care (Appleby and Devlin 2004; Dawson et al. 2010). Ideally, PROMs data are collected before and after healthcare service use, so that *change* in patient-reported outcomes (PROs), attributable to the service or intervention received, can be identified (Appleby and Devlin 2004; Dawson et al. 2010). This provides an assessment of whether the patient feels better, and how much better. A combination of a generic and a condition-specific or speciality-specific PROM is considered optimal because, although the latter have greater face validity and credibility, generic PROMs enable comparisons across conditions (Black 2013; Devlin and Appleby 2010). It has been suggested that PROMs data could be used to transform how healthcare is organised and delivered (Black 2013), and to manage healthcare performance by feeding PROMs data back to the providers (Appleby and Devlin 2004; Haywood et al. 2006). In a related vein, healthcare teams are beginning to use PROMs data to improve the quality of care (Boyce et al. 2014). However, despite evidence that PROMs use is acceptable to patients, can enable earlier symptom detection, and may improve clinician-patient communication, there is little understanding regarding how this may occur, and definite evidence that PROMs data can be used by clinicians to achieve service quality improvements that improve PROs and other outcomes is lacking (Chen et al. 2013; Howell et al. 2015). Nevertheless, there has been some suggestion that PROMs data, used as a management tool with specialized patient populations in an outpatient setting, could result in better outcomes for patients (Boyce and Browne 2013). As a consequence there may be some benefit in exploring this approach in a UK genetic counseling context, where it is delivered as part of specialized outpatient care.

The aim of this study is to explore the benefits of, and challenges to, using PROMs for service quality improvement in clinical genetics. This is achieved through a case-study of a local clinician-led service quality improvement initiative. The PROMs chosen were the generic Euroqol (EQ-5D) (Euroqol Group 2009) and the clinical genetics-specific Genetic Counseling Outcome Scale (GCOS-24) (McAllister et al. 2011), along with an audit tool (Skirton et al. 2005). Of particular note is the use of GCOS-24 in service evaluation exercises in six of the 25 UK regional clinical genetics centres in 2011–12 (McAllister 2016). Findings demonstrated that (i) NHS clinical genetics services can deliver significant measurable patient benefits and (ii) GCOS-24 has potential to generate information about routine NHS clinical genetics practice that may be useful to decision-makers (McAllister 2016). However, these exercises also identified the need for significant further work including the need to develop an approach to support clinical teams to use PROMs data effectively to contribute to a continuous quality improvement cycle focused on optimising patient benefits (McAllister 2016), as explored here.

## Methods

A combination of quantitative and qualitative methods was used. All procedures followed were in accordance with the ethical standards of the responsible committees on human experimentation (institutional and national) and with the Helsinki Declaration of 1975, as revised in 2000 (5). Informed consent was obtained from all patients for being included in the study.

### Quantitative Instrumentation

Three quantitative data collection instruments were utilized in the study. First, EQ-5D (See Supplement A) was chosen because it is the generic PROM favoured by the UK National Institute for Health and Excellence (NICE), a public body of the DH whose remit is evaluation of the efficacy and cost-effectiveness of interventions carried out by the NHS (NICE 2014). EQ-5D is favoured by NICE because it can be used to calculate Quality Adjusted Life Years and can therefore be used in economic evaluations of health interventions (Phillips 2009). EQ-5D captures five domains of health: mobility, self-care, usual activities, pain/discomfort and anxiety/depression (Euroqol Group 2009). The minimum score for EQ-5D is 5 and the maximum score is 25.

Second, GCOS-24 was chosen because it is a well-validated clinical genetics-specific PROM with proven validity, reliability and responsiveness to change over time (McAllister et al. 2011) (See Supplement B). GCOS-24 captures empowerment, a construct summarising patient benefits from using clinical genetics services (genetic testing and counseling) comprising five dimensions: cognitive control (having sufficient knowledge and understanding about the condition, signs and symptoms, implications and risks to self and other relatives, as well as knowing what support is available); decisional control (having options or feeling able to make informed decisions for managing genetic risk); behavioural control (feeling able to actively use health and social services effectively to reduce harm or improve life situations for self, children and/or at-risk relatives); emotional regulation (feeling able to effectively manage the emotional effects of genetic diseases, including coping and adjustment) and; hope (feeling able to look to the future with hope for a fulfilling family life for oneself, relatives and/or future descendants).

Third, the audit tool was chosen because, although it cannot be used to measure pre/post *change* (because of the way the items are worded), it is familiar to clinicians in the service (See Supplement C). It is referred to by AWMGS staff as the ‘AWMGS satisfaction questionnaire’, although it might be more appropriately referred to as a patient-reported experience measure. The audit tool addresses six outcome areas (Skirton et al. 2005), noted here with sample items: enhanced understanding (e.g. I have more understanding of what causes the condition); positive psychological change (e.g. I have greater peace of mind); respect for autonomy (e.g. My main questions were answered); adaptation (e.g. I am more able to ask for help if I need it); disequilibirium (e.g. I couldn’t understand what I was told); and value of contact (e.g. I felt treated as an individual). Responses were measured on a seven point scale, indicating strength of (dis)agreement.

### Quantitative Participants, Procedures and Analysis

Following governance approval by the relevant hospital Research and Development offices, quantitative PROMs data were collected from patients before and after clinic attendance, using GCOS-24 and EQ-5D 5-level version (Herdman et al. 2011; Janssen et al. 2013). These were mailed to all patients having a first appointment at AWMGS between February and July 2015 using approved informed consent procedures, and enclosing a pre-paid envelope for return of the completed PROMs pack. No demographic data were collected. If the patient referred to the service was a child, his/her parent was asked to complete the PROMs. Parents can be considered ‘patients’ in clinical genetics, as they may be at risk for having an affected child in subsequent pregnancies. Following clinic attendance, all patients who had completed and returned GCOS-24 and EQ-5D before clinic attendance were sent a second PROMs pack comprising GCOS-24, EQ-5D and the audit tool (as previously noted, the wording of the questions within this tool does not enable pre/post intervention evaluation). PROMs responses were input into SPSS for Windows. Data were analysed using descriptive statistics and repeated measures analysis of variance. A Bonferroni correction was used for multiple comparisons.

### Communication with the Clinical Team

It was important that the clinical team was engaged with and kept up to date with the process, and that discussions took place about how the PROMs data might be useful. Communication began with a seminar in September 2014 describing the plan for the study. The evaluation team met with eight AWMGS clinicians, who had expressed an interest in participating in the study, once every two months during the data collection period (February–July 2015) to monitor progress. At these meetings, patient response rates were monitored, and discussions took place about how the PROMs data might be useful.

### Qualitative Participants, Procedures, Instrumentation and Analysis

Following ethics approval from Cardiff University School of Medicine, the eight AWMGS clinicians involved in the study were invited to participate in an interview (using approved informed consent procedures) to explore their perceptions about the value and feasibility of using the PROMs data for service quality improvement. The aim of qualitative research is to explore the perspectives of human participants and it can be used to gain an in-depth understanding of people’s views about a given phenomenon or activity (Wengraf 2004). Qualitative methods are suitable for exploratory research in areas which are not entirely understood, where the participants are actively involved in the generation of new healthcare methods, and where some relevant variables have not yet been fully identified (Wengraf 2004). As the purpose of this research was to explore for the first time the perspectives of AWMGS clinicians about the usefulness of PROMs data for service quality improvement, a qualitative approach was appropriate. Individual, face-to-face, semi-structured interviews with participants were conducted. The interview guide (see Supplement D) was developed by MM, and approved by the National Institute for Social Care Health Research Permissions Coordinating Unit (NISCHR PCU). ACT conducted all the interviews and confirmed written consent from all participants at the beginning of each interview.

Interviews were audio-recorded, transcribed in full and analysed using thematic analysis, which permits identification, analysis and reporting of patterns (themes) that appear in qualitative data in order to identify common elements across the interviews. Whilst the interview schedule was thematically structured it was exploratory in orientation. Thus, the focus on challenges and benefits inherent in the research question meant that analysis took account of themes across the sections of the interview schedule. Data were thematically analysed by the first author to ensure internal consistency. Emergent themes were discussed and validated with the last author. The analysis began by comparing the transcripts with the audio-recording to check that they were correctly transcribed and familiarisation with the transcripts by reading them several times. During this process, notes were taken about initial ideas raised by the respondents relating to the research question. As per Gioia et al. (2013), this initial stage involved identifying first-order concepts, adhering to respondents’ terms. The next stage involved developing second-order themes, used as initial codes, by grouping respondents’ terms into thematically oriented meaning groups. As many data as possible were codified, to enable the identification of third-order aggregate dimensions (e.g. quality improvement, patient-centred service), premised on classifying the codes into larger groups (Gioia et al. 2013). These overarching findings were then checked for relevance and consistency across the dataset, as well as informativeness in relation to the research question. This enabled refinement and further specification of the aggregate dimensions.

## Results

### Quantitative Component

PROMs packs were mailed to 926 patients prior to clinic attendance, and 213 completed PROMs packs were returned, giving an initial response rate of 23%. Of the 213 patients who returned a pre-clinic PROMs pack, 96 (45%) also returned a post-clinic PROMs pack. This resulted in 96/926 usable responses, premised on matched pre and post clinic PROMs packs, giving a final response rate of 10.3%. Statistically significant improvement in GCOS-24 scores following clinic attendance (*p* < 0.05), with a medium-to-large effect size (Cohen’s d = 0.64), demonstrated that patient benefits were delivered. The mean pre-clinic GCOS-24 score was 104.45 (SD = 16.5), and the mean post-clinic GCOS-24 score was 115.33 (SD = 17.6). There was no significant difference in the pre appointment questionnaire scores for those only responding to the pre appointment questionnaire (mean = 105.41, SD = 16.81) and those who responded to both (mean = 104.45 SD = 16.544); t(200) = .405, *p* = .686. A sub scale analysis was carried out using repeated measures analysis of variance, comparing pre and post appointment GCOS-24 scores for the seven sub-scales. This analysis demonstrated that there was a significant improvement in post appointment GCOS-24 scores for the hope, support, family impact, powerlessness, referral clarity and adaptation sub-scales but no significant difference in the emotional regulation sub-scale (See Table 2).

High post-clinic scores on the audit tool (mean = 95.5; highest possible score = 126) also demonstrated good outcomes, although there were no pre-clinic scores to compare with. However, there was no significant change in EQ-5D scores. The mean pre-clinic EQ-5D score was 21.61, and the mean post-clinic score was 21.25 (*p* = 0.269).

### Communication with the Clinical Team

Discussions with the clinical team focussed initially on the disappointing response rates. A number of approaches to increasing the response rate in future exercises were discussed e.g. PROMs to be made available in the waiting room prior to appointments, and providing access to online versions of the PROMs that patients could complete using PCs, tablets and smartphones. Discussions increasingly focussed on how the PROMs data could be useful. Members of the clinical team were not surprised by the lack of improvement in scores seen on the EQ-5D, and their views on this are reported in the qualitative analysis below. These data were not felt to be useful for quality improvement. The team was pleased by the GCOS-24 overall change scores, and by the scores on the audit tool, and these were perceived as cause for celebration. The group agreed that it was useful to consider the overall sub-scale scores. However, they were also keen to look at change scores on individual GCOS-24 items to obtain a more detailed insight into where patients were deriving greater and lesser benefit. The GCOS-24 was felt to be most useful because these data showed *change* in scores following clinic attendance, which the audit tool did not show. GCOS-24 change scores on individual GCOS-24 items following clinic attendance are shown in Table 2.

In keeping with the overall sub-scale scores, Table 2 demonstrates improvements in patients’ understanding of their condition, their understanding of implications for themselves and (specified) relatives, knowledge of where to seek further help (educational, financial, social support) and their feeling of hope and ability to make plans for the future. Table 2 also shows that patients are achieving fewer improvements in feelings of distress, guilt, powerlessness, ability to cope and hope for their children’s future. Consideration of the individual items provides a little more insight than the sub-scale analysis, which demonstrated lack of improvement in the emotional regulation sub-scale only. These changes were achieved following just one appointment, so any improvement is noteworthy.

### Qualitative Component

Of the eight AWMGS health professionals who were invited to participate, six agreed to be interviewed. Two participants were consultants in clinical genetics (MDs in the UK context) and four were genetic counselors (See Table 1). Recruitment and interviews were carried out in May–June 2015. Although the number of interviewees was small, some suggest that between six and eight (Kuzel 1992) or six and twelve (Guest et al. 2006) participants can be sufficient for relatively homogeneous populations (in this case, longstanding departmental colleagues). However, we acknowledge the benefits of larger numbers to substantiate data saturation, and the need for follow-on research from this exploratory study. Nonetheless, thematic consistency was evident, with no new major themes emerging in the final interview. Three main themes were identified with subthemes in each: *quality improvement (QI)*, *patient-centered service*, and *use of PROMs*.

**Table 1:** Sample characteristics

Participant number	Type of genetics health professional	Length of service in clinical genetics	Length of service in AWMGS	Area of specialism
1	Genetic counselor	8 years	5 years	Cancer genetics
2	Genetic counselor	1 year and 3 months	3 months	General genetics
3	Clinical geneticist	25 years	25 years	General and cancer genetics
4	Genetic counselor	3 years	1 year	General genetics
5	Clinical geneticist	8 years	8 years	General genetics
6	Genetic counselor	20 years	20 years	General genetics

#### Quality Improvement (QI)

All participants agreed that in order to assess whether the service offered is of good quality, it is not sufficient to assess only process measures (e.g. waiting times). It is more important to evaluate the benefits obtained directly by patients, using a measure that could capture patients’ perceptions before and after using the service. Participants also highlighted that the NHS is implementing use of PROMs to evaluate service quality in other specialties. They also reported that evaluation of CGS has focussed on process issues such as patient complaints and compliments as well as waiting times, but they argued that these measures provide no insight into whether patient benefits are delivered (Table 2).

The ones that we’ve commonly used like patient compliments, patient complaints, don’t really give a very good overall view of how you’re doing (ppt 5)

…to have some sort of measure of outcome because without a measure of outcome, just dealing with satisfaction afterwards, people um, it’s not so helpful in deciding what effect you have actually had (ppt 3)

It’s also probably institutionally necessary for us to use them increasingly because we are likely to be required to do so, to use some sort of patient-reported outcome measure (ppt 3).

**Table 2:** The Genetic Counseling Outcome Scale (GCOS-24): Change scores for GCOS-24 items and sub-scales

Item number & wording (McAllister et al. 2011)	Mean difference in score	Significance^b^ (2-tailed, * *p* < 0.05)	Sub-scale	Mean difference in score	Significance^b^ (2-tailed, * *p* < 0.05)
8. I feel positive about the future.	0.344	0.024*	Hope	1.281	0.007*
9. I am able to cope with having this condition in my family.	0.198	0.247			
19. I am hopeful that my children can look forward to a rewarding family life.	0.271	0.167			
20. I am able to make plans for the future.	0.469	0.005*			
2. I can explain what the condition means to people in my family who may need to know.	0.479	0.003*	Support	3.094	0.000*
5. #I don’t know where to go to get the medical help I / my family need(s).	0.677	.008*			
10. #I don’t know what could be gained from each of the options available to me.	0.573	0.007*			
15. I know how to get the non-medical help I / my family needs (e.g. educational, financial, social support).	0.688	0.003*			
16. I can explain what the condition means to people outside my family who may need to know (e.g. teachers, social workers).	0.677	0.001*			
4. #When I think about the condition in my family, I get upset.	0.156	0.443	Emotional regulation	0.698	0.123*
11. #Having this condition in my family makes me feel anxious.	0.260	0.177			
21. #I feel guilty because I (might have) passed this condition on to my children.	0.281	0.193			
3. I understand the impact of the condition on my child(ren)/any child I may have.	0.531	0.020*	Family impact	1.896	0.001*
12. #I don’t know if this condition could affect my other relatives (brothers, sisters, aunts, uncles, cousins).	0.740	0.006*			
18. #I don’t know who else in my family might be at risk for this condition.	0.625	0.015*			
13. #In relation to the condition in my family, nothing I decide will change the future for my children / any children I might have.	0.073	0.723	Powerless-ness	1.052	0.032*
17. #I don’t know what I can do to change how this condition affects me / my children.	0.760	0.001*			
22. #I am powerless to do anything about this condition in my family.	0.219	0.332			
1. I am clear in my own mind why I am attending the clinical genetics service.	0.365	0.030*	Referral clarity	1.292	0.004*
14. I understand the reasons why my doctor referred me to the clinical genetics service.	0.469	0.013*			
23. I understand what concerns brought me to the clinical genetics service.	0.458	0.007*			
6. #§I can’t see that any good things have come from having this condition in my family.	0.438	0.022*	Adaptation	1.229	0.001*
7. I can control how this condition affects my family.	0.323	0.097			
24. I can make decisions about the condition that may change my child(ren)‘s future / the future of any child(ren) I may have.	0.469	0.027*			

Based on estimated marginal means. Bold indicates *p* < 0.05

^b^Adjustment for multiple comparisons: Bonferroni

*The mean difference is significant at the 0.05 level

# Items reverse coded for analysis

§ Item wording reversed at the request of the clinical team from original wording ‘I can see that good things have come from having this condition in my family’

In supporting quality improvement, participants noted that having additional detail regarding service users’ condition, reason for referral, and personal characteristics would enhance the utility of the data, and scope to tailor amendments in service provision.

So I think people’s expectation levels, depending upon the client group, is going to be different and I’m sure that will, or may, reflect in the answers in terms of their satisfaction. So I think that’s probably an important part of the analysis, if you’re going to be able to identify which patient group the respondents came from. (ppt 6)

Maybe it’s different in different appointments, different conditions, or whatever, and then hopefully being able to target, you know, whether perhaps in cancer sessions maybe we spend too much time doing information and we need to devote more time to psycho-social. (ppt 1)

#### Patient-Centred Service

In all interviews, the concept of patient-centeredness was one of the strongest emerging themes either directly or indirectly. Participants reported that the service itself and its quality should be focused on the benefits and needs of AWMGS patients. Participants explained that collecting PROMs data could enable them to ensure the best possible patient experiences and outcomes, thus ensuring patient-centred care. Participants considered themselves to be patient-centered health professionals, and also considered the administrative team to be part of, and have invested in providing, a fully patient-centered service.

We’re providing this service *for the patients* (ppt 2)

(the administrative team) are very patient-centred, you know, they want to do the best for the patients that we have coming into the clinical genetics service. (ppt 5)

By using these patient reported outcomes we can check that we are actually meeting what they need from the service and therefore hopefully deliver the best service that we can […] the professionals here […] want to give the best service they possibly can to patients and therefore they are […] very willing to engage with quality improvements and with things that they can do to ensure that they’re meeting patient needs (ppt 1)

#### Use of PROMs

All participants considered the use of PROMs appropriate and necessary to evaluate patient outcomes, as well as a beneficial tool to enable them to think about service improvements. GCOS-24 was considered useful by the participants because it was validated specifically to evaluate genetic counseling services, designed through a systematic and logical procedure taking account of CGS professionals’ and patients’ perspectives. GCOS-24 was also valued because it could be applied before and after the service to observe score changes.

It’s the one that’s been validated for genetic counseling specifically, and therefore it seems to me to be a good way to measure what we are trying to do (ppt 1).

And those things are really specific to the genetic counseling service and relevant and can be applied before and afterwards. (ppt 2)

… the questions repeated after the event so one would hope that their expectations have been fulfilled, their questions answered, hopefully their level of anxieties reduced and their knowledge level has increased (ppt 6).

Participants also considered it important and useful to evaluate patients’ experiences using the audit tool, when applied together with a PROM. Participants appreciated the audit tool because it was developed by a genetic counselor, and because it includes some open questions.

(The audit tool) does cover some information and in general we have found that patients have been mostly satisfied. But that doesn’t tell us if we are really necessarily meeting the needs of the patients more specifically than that and so by using these patient reported outcomes we can check that we are actually meeting what they need from the service (ppt 1).

Well it helps that it was made by somebody who’s familiar with the genetic counseling service. So I think this would be a good one to use as well. (ppt 2)

So satisfaction questionnaires have their place but not to look at the impact of what you have done in the clinic. (ppt 3)

All participants reported that they perceived EQ-5D as not useful to evaluate patient benefits from attending AWMGS. Specifically, participants remarked that in general, their patients are physically well and therefore they considered most constructs captured by EQ-5D irrelevant.

… about activities of daily living, I think that’s completely irrelevant to it because most of our patients are well (…) actually we don’t manage those difficulties so it’s not relevant. (ppt 5)

The vast majority of our patients are kind of not ill, they’re walking. So I think a lot of the questions are not necessarily particularly relevant. (ppt 6)

Moreover, four of the participants explained that for patients, it could be potentially confusing to be asked the EQ-5D questions, because patients might think that these questions are inappropriate for the type of condition they have.

I think people will be confused a bit about the EQ5D because it’s, you know, not relevant to genetics counseling about whether they can wash and dress themselves isn’t something we’re really concerned, well you know that’s not to do with the treatments we provide really. (ppt 4)

Nevertheless, most participants considered it necessary to include EQ-5D in the PROMs set for this initiative because it is the generic PROM preferred by NICE and to demonstrate that it might not be useful for evaluating CGS. One participant was concerned that using EQ-5D could make the CGS vulnerable. In the future the NHS could allocate CGS funding on the basis of patients’ EQ-5D scores, and the participant was concerned that if a CGS did not show benefits using EQ-5D, this could impact on future funding.

It’s important to include it to demonstrate that perhaps that may not be the most useful one. (ppt 1)

So that could make us vulnerable. So if the health service adopted the position that they exist to maximise people’s EQ-5D scores then they would not want to fund genetics very much because we probably have little if any impact on EQ-5D. They would put the money into treating arthritis, heart disease. (ppt 3)

Some participants voiced concerns that collecting and reporting PROMs data might be experienced as threatening both for the service and for some members of the clinical team:

Everybody wants the scores to be good and to reflect well on their practice […] a challenge could be that it might be the opposite. So I guess there’s that challenge of “Ooh, our practice has been looked at, and what if comes up wanting?” (ppt 6)

I think that it is sometimes difficult to get negative results, I suppose personally and as a service. And I think that’s a challenge for people, you know, this is very new to us and I think so that is difficult, you know, to get people to not take it personally but to think more that this is an area where we can improve. (ppt5)

The PROMs were perceived by participants as being generally patient-friendly and easy to understand. There was also some concern voiced about the challenges that some patients might have in completing the PROMs (e.g. patient with learning disability), but they also mentioned possible solutions. For example, patients with learning disabilities could be afforded support in answering the questionnaire, as described below.

if your patient has any degree of learning disability then I think it becomes much harder so I think someone who struggles with reading and writing would find it hard […] for someone else, to go through the questionnaire with them and make sure, talk about each question and be clear that they wanted to give a good score or not so good score […] It’s better if it’s an independent person if possible.

Regarding patient response rates, which were disappointing, participants were concerned that not all the patients had the same availability or willingness to respond to questionnaires. As a result, the returned data might not be representative of all patients to whom the service was delivered. Participants suggested that one way to increase patient participation was to allow patients to respond to the questionnaires using email or online questionnaires. Other proposed solutions were to make the questionnaires available in clinic waiting rooms on paper or using a tablet computer (iPad), or for the administrative (admin) team to telephone patients to remind them about the option to participate in the initiative.

I think to improve how you get it people have to be able to do it instantaneously. So I do wonder, with newer IT in mind, emailing questionnaires, you know, or a survey that they can click straight to and fill in, you know, like Survey Monkey, having you know IT where you can ask and say, you know, “Would you mind going onto this iPad?” (ppt 5)

[…] perhaps these could be given out in the clinic setting or prior to the clinic or telephone contact be made. (ppt 6)

Participants considered that the clinical team is best placed to interpret the data, and brainstorm potential quality improvement (QI) activities to address areas where the service could be delivering greater patient benefits. They felt that the best approach was for a small sub-group of the service’s clinical staff to tackle the task of data interpretation, and then present it to the full group in a meeting, for discussion of possible issues that could be addressed using QI initiatives.

Once we’ve got the information you know from this project, whether it would be perhaps that a smaller group looks at it in the first place and comes up with some suggestions for how we can do the quality improvement, and then taking that to the whole service and getting people’s buy-in. (ppt 1)

So within that small group that would be, you know that’s manageable, and then if we do suggest any changes we’d have to go out to the wider team to discuss implementation or you know how we’re going to bring about those changes. (ppt 4)

Most participants said that the meetings should be regular but flexible (approximately every 3–6 months), allowing for periods of extra need or when data have not dramatically changed. Regarding QI initiatives, there was no clear consensus about how to address lack of significant mean improvement in areas relating to patient’s reported emotional regulation. Some participants advocated more clinic time to use their counseling skills:

[…] the shorter an appointment is the less counseling skills you can do, you know, you have to get through so much information as well. (ppt 4)

Others advocated more counseling skills training:

And although we’ve had counseling skills training I think there’s probably more work we could do, so better training. (ppt 1)

Participants would like to use PROMs data in a cyclical way e.g. Plan-Do-Study-Act cycles, with regular collection and analysis of PROMs data, with appropriate steps taken to address areas where patients are not benefitting as much as they could be:

just getting everybody together, discussing the results, discussing how we can make these results even better for next time, and then hopefully re-doing it, like an audit, re-doing the questionnaires with a different group of patients and comparing whether the improvement, or if there is an improvement first of all, and then if their improvement is better than last time. (ppt 2)

There was no clear consensus on how the team could best address areas where patients were deriving significantly less benefit (patients’ feelings of distress, guilt and powerlessness). Access to training in brief counseling interventions was made available to the clinical team as a way of developing new skills in this area, with a plan to collect further PROMs data 18 months later. However, the significance of achieving discernible difference in many PROMs items based on a single consultation needs to be reiterated.

## Discussion

This study is the first study to explore usefulness of PROMs data for quality improvement in clinical genetics services. Findings demonstrate that PROMs were considered by AWMGS health professionals who participated in the study to be a good tool to measure the benefits to patients from using the CGS, enabling direct capture of patients’ change scores following clinic attendance. Of the questionnaires used in this initiative, GCOS-24 and the audit tool were considered useful and EQ-5D was considered significantly less useful for evaluation of CGS. The main barrier encountered to effectively using PROMs data for service QI was poor patient response rates.

Because this is the first study exploring usefulness of PROMs data for quality improvement in clinical genetics services, findings cannot be directly compared with previous studies. However, GCOS-24 was recently used in Vancouver for evaluation of a psychiatric genetic counseling service (Inglis et al. 2015). Although the Vancouver study was a service evaluation without a quality improvement component and did not seek to explore the perspectives of the professionals involved, the Canadian study also showed a significant improvement in GCOS-24 scores after a genetic counseling consultation, demonstrating usefulness of GCOS 24 for service evaluation.

Regarding EQ-5D, although it has never before been reported to evaluate CGS, this study is not the first time that this PROM was considered unhelpful for evaluating a service because that service addresses patient outcomes that are not captured by EQ-5D (Phillips 2009; Wailoo et al. 2010). However, without EQ-5D, it is not possible to calculate QALYs delivered to patients of CGS, limiting use of PROMs in economic evaluations of CGS.

Findings in the current study support findings in a previous study exploring healthcare professionals’ perspectives of using PROMs to evaluate other healthcare services. Specifically, a review of qualitative studies exploring healthcare professionals’ views of using PROMs data to improve healthcare quality (Boyce et al. 2014) found that participants appreciated PROMs as a tool to complement their own clinical judgment and encouraged their professional development, a sentiment also expressed by respondents in the present study. One concern reported in the Boyce et al. (2014) review is the possible use of PROMs data to justify cost-cutting. Similarly, one participant in the present study expressed concern that the NHS might use this information to justify cutting CGS resources. However, the present study also demonstrated commitment amongst AWMGS clinical staff to using PROMs data for the benefit of patients and for their own professional development. The qualitative aspects of this exploratory study also signal the importance of further exploring the challenges faced by individual employees, who reported responding to PROMs feedback on a personal, as well as professional level. Furthermore, the finding that patients derived little benefit in the emotional regulation sub-scale of GCOS-24, and the additional information from the analysis of individual GCOS-24 items regarding fewer improvements in feelings of distress, guilt, powerlessness, ability to cope and hope for their children’s future, may signal the need for additional investment in counseling to address deeper levels of emotional distress. GCOS-24 could be a useful tool to evaluate any marginal benefit of additional counseling sessions.

### Study Limitations

The major limitation of this study is the low patient PROMs response rate. The present study is not the first to report difficulties in collecting PROMs data as a barrier to using PROMs for service evaluation (Dunckley et al. 2005; Meehan et al. 2006; Slater and Freeman 2005). Other initiatives collecting PROMs data routinely for service evaluation report the time required for workers to input the data on computers as problematic (Abernethy et al. 2008). In the AWMGS study, this task along with PROMs data analysis was completed by MSc in Genetic Counseling students (DF & CT), and so these tasks did not require completion by a member of AWMGS team. Participants suggested online data collection and analysis may be helpful in the future. However, these novel methods would require investment. Studies show that the use of IT for PROMs data collection (such as tablet computers in outpatient clinics) is feasible and acceptable (Bennett et al. 2012; Tavabie and Tavabie 2009). This could potentially mitigate the low response rates. In addition, as data were collected and discussed by the team, it became apparent that demographic data would have been extremely helpful. Unfortunately, the governance arrangements put in place at the outset of the study precluded their addition. There is scope for future research to consider whether the types of benefits derived by patients vary according to their condition, reason for referral (e.g. families referred for paediatric versus adult onset conditions; cancer genetic testing; carrier testing), or personal characteristics. Furthermore, it is as yet unclear how much change in GCOS-24 scores is important and meaningful to patients. It will be important to establish the Minimum Clinically Important Difference (MCID) (King 2011) for the GCOS-24, which is the smallest change in a treatment outcome that matters to patients, or that patients would identify as important. This will be useful for interpreting GCOS-24 scores in future research, service evaluation and quality improvement work.

A number of studies in other healthcare specialties (Appleby and Devlin 2004; Dunckley et al. 2005; Hughes et al. 2004; Meehan et al. 2006; Slater and Freeman 2005) reported that a lack of collaboration between colleagues using PROMs could be a barrier to effective implementation of routine PROMs assessment. However, this did not emerge in the AWMGS initiative; participants considered both themselves and the administrative staff very much engaged in the PROMs data collection exercise, and as sharing the goals of wanting to provide patient-centered care. Nevertheless, only a proportion of AWMGS clinical staff participated in this study, and if PROMs assessment should become routine, it may be worth monitoring whether all staff continue to be invested and committed to this initiative (and the factors that enable this), since staff involvement was considered by participants to be fundamental to success.

A second limitation of this study is that the sample size was also small in the qualitative study, and although it may represent the views of AWMGS clinicians, it is not necessarily representative of CGS more widely. So while the themes that emerged from the analysis are generally concordant with what is reported in the literature, they cannot be extrapolated to other CGS beyond AWMGS. However themes are still meaningful because the present sample represents a majority of AWMGS clinical staff involved in the PROMs QI initiative, including both clinical geneticists and genetic counselors.

A third limitation of this study is the threat to internal validity that is posed due to the lack of a comparison group who did not attend the clinic. This study was designed as a before-after comparison, with the threat to internal validity mitigated since GCOS-24 has established test-retest reliability (*r* = 0.86) and responsiveness following attendance at a UK clinical genetics service (effect size of Cohen’s d = 0. 70) (McAllister et al. 2011).

Despite these limitations, this study also has strengths. Thematic consistency was evident across the interviews. The context in which this project was developed is innovative for using PROMs in CGS, and for comparing measures to other assessments, notably the EQ-5D and GCOS-24. Although PROMs (including GCOS-24) have been used for service evaluation in CGS (Inglis et al. 2015), this is the first reported example of PROMs data being used for quality improvement in CGS. Furthermore, this study is the first to explore CGS professionals’ views about using PROMs for continuous evaluation and QI of the service.

### Practice Implications

Measuring PROMs routinely in clinical genetics practice and sharing data through staff forums focused on discussing findings and reflecting on any appropriate action to improve practice has potential to improve patient-centred care and maximise patient benefits. Although this requires giving staff space and time to step out of clinical activities, the participants in this study found the exercise to be both rewarding and useful. It represented an opportunity to celebrate areas of practice where patients were gaining benefits, as well as identify some areas where improvements could be made. As noted above, development of online methods for data capture and automated data analysis is likely to maximise patient response rates to PROMs.

### Research Recommendations

Future research should take account of whether and how reason for referral or demographic characteristics influence patient outcomes as measured by PROMs data. Futhermore, additional research exploring factors supporting uptake of PROMs amongst clinical genetics patients would be helpful to establish whether patients consider PROMs to be a useful way to provide feedback to clinical services, and what processes could help to maximise PROMs response rates. It would also be helpful to explore with clinical teams what improvement skills they require to effect improvement, based on PROMs feedback. This could identify potential training needs and strategies for organisational support and, in time, potential additions to education curricula.

The clinical team and the research team were pleased that improvement was evident following only one appointment at the clinical genetics service. It would be interesting for future research to evaluate the marginal benefit of additional counseling sessions, and the tipping point for emotional benefit. Establishing the Minimum Clinically Important Difference (MCID) (King 2011) for the GCOS-24, will be useful for interpreting GCOS-24 scores in future research, service evaluation and quality improvement work, and for making a case for further investment in genetic counseling.

The present study explored usefulness of EQ-5D for evaluation of clinical genetics services, but this PROM was not found to be useful. EQ-5D is favoured by NICE in the UK because it has available preference weights to enable its use in economic evaluations. For PROMs to be useful in economic evaluations of clinical genetics services, it will be important to develop a suitable preference-based PROM. Although GCOS-24 has good psychometric properties (reliability, validity, responsiveness), it is currently unsuitable for use as a measure of patient benefit in economic evaluations because it is too unwieldy (long) with no available preference weights. One approach to address this could be to identify five or six GCOS-24 items and levels for valuation (creating GCOS-short), and use health economics methods to identify and explicitly incorporate preference weights to reflect the relative importance that people place on the specific GCOS-short items and levels, and for these weights to be reflected in summary measures. This would facilitate future work to compare alternative clinical genetics interventions aimed at promoting patient empowerment, to better take account of the relative benefits of the interventions for economic evaluation. It will also be important to continue to work to identify a suitable generic PROM for use in clinical genetics, since EQ-5D was found not to be useful. ICECAP-Adult may provide a useful alternative to EQ-5D (Al-Janabi et al. 2012; Sen 2007). ICECAP-Adult was designed to capture the concept ‘capability’ for use in economic evaluation, and comprises five domains: attachment (ability to have love, friendship and support), stability (ability to feel settled and secure), achievement (an ability to achieve and progress in life), enjoyment (ability to experience enjoyment and pleasure), and autonomy (ability to be independent) (Al-Janabi et al. 2012; Sen 2007).

## Conclusions

This study has demonstrated that use of PROMs, and in particular GCOS-24, was viewed positively by the AWMGS participants for service evaluation and quality improvement. It also demonstrates the general desire of AWMGS staff to evaluate the service using PROMs routinely for QI. Over time it will be important to demonstrate whether patients obtain greater benefit from the possible improvements implemented in the service based on the PRO data by repeating the initiative. PROMs data will continue to be collected regularly by AWMGS, with members of the clinical team analysing and reporting on the success or otherwise of implementing additional training in brief counseling interventions. This study has also identified potential enablers for using PROMs data for quality improvement such as online data collection and automated data analysis, although these initiatives will require initial investment.

## Electronic supplementary material

ESM 1 (PDF 121 kb)

ESM 2 (DOCX 46 kb)

ESM 3 (DOCX 44 kb)

ESM 4 (DOCX 17 kb)
