# Supplementary material for: Using Patient-Reported Outcome Measures for Quality Improvement in Clinical Genetics: an Exploratory Study
Source: J Genet Couns. 2017 Mar 9;26(5):1017–28. doi: 10.1007/s10897-017-0079-6 (PMC5582073; doi:10.1007/s10897-017-0079-6)
Supplement: Supplementary file 1 — (PDF 121 kb) [file 10897_2017_79_MOESM1_ESM.pdf]

EQ-5D-5L

Health Questionnaire

English version for the UK

Sample

UK (English) © 2009 EuroQol Group EQ-5D™ is a trade mark of the EuroQol Group

Under each heading, please tick the ONE box that best describes your health TODAY.

MOBILITY

I have no problems in walking about	❑
I have slight problems in walking about	❑
I have moderate problems in walking about	❑
I have severe problems in walking about	❑
I am unable to walk about	❑

SELF-CARE

I have no problems washing or dressing myself	❑
I have slight problems washing or dressing myself	❑
I have moderate problems washing or dressing myself	❑
I have severe problems washing or dressing myself	❑
I am unable to wash or dress myself	❑

USUAL ACTIVITIES *(e.g. work, study, housework, family or leisure activities)*

I have no problems doing my usual activities	❑
I have slight problems doing my usual activities	❑
I have moderate problems doing my usual activities	❑
I have severe problems doing my usual activities	❑
I am unable to do my usual activities	❑

PAIN / DISCOMFORT

I have no pain or discomfort	❑
I have slight pain or discomfort	❑
I have moderate pain or discomfort	❑
I have severe pain or discomfort	❑
I have extreme pain or discomfort	❑

ANXIETY / DEPRESSION

I am not anxious or depressed	❑
I am slightly anxious or depressed	❑
I am moderately anxious or depressed	❑
I am severely anxious or depressed	❑
I am extremely anxious or depressed	❑

Sample

UK (English) © 2009 EuroQol Group EQ-5D™ is a trade mark of the EuroQol Group

- We would like to know how good or bad your health is TODAY.
- This scale is numbered from 0 to 100.
- 100 means the <u>best</u> health you can imagine.
  0 means the <u>worst</u> health you can imagine.
- Mark an X on the scale to indicate how your health is TODAY.
- Now, please write the number you marked on the scale in the box below.

YOUR HEALTH TODAY = ☐

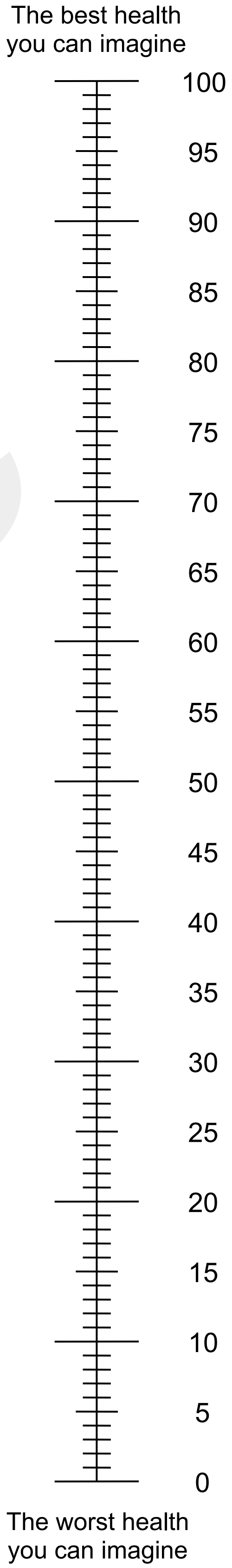

Sample

UK (English) © 2009 EuroQol Group EQ-5D™ is a trade mark of the EuroQol Group
