# Supplementary material for: Using Patient-Reported Outcome Measures for Quality Improvement in Clinical Genetics: an Exploratory Study
Source: J Genet Couns. 2017 Mar 9;26(5):1017–28. doi: 10.1007/s10897-017-0079-6 (PMC5582073; doi:10.1007/s10897-017-0079-6)
Supplement: Supplementary file 2 — (DOCX 46 kb) [file 10897_2017_79_MOESM2_ESM.docx]

Supplement B: The Genetic Counseling Outcome Scale (GCOS-24)

# Using the scale below, circle a number next to each statement to indicate how much you agree with the statement. Please answer all the questions. For questions that are not applicable to you, please choose option 4 (neither agree nor disagree).

agree

slightly disagree

neither agree nor disagree

slightly agree

disagree

strongly agree

strongly disagree

1 = strongly disagree

2 = disagree

3 = slightly disagree

4 = neither disagree nor agree

5 = slightly agree

6 = agree

7 = strongly agree

| 1 | I am clear in my own mind why I am attending the clinical genetics service. | 1 | 2 | 3 | 4 | 5 | 6 | 7 |
| --- | --- | --- | --- | --- | --- | --- | --- | --- |
| 2 | I can explain what the condition means to people in my family who may need to know. | 1 | 2 | 3 | 4 | 5 | 6 | 7 |
| 3 | I understand the impact of the condition on my child(ren)/any child I may have. | 1 | 2 | 3 | 4 | 5 | 6 | 7 |
| 4 | When I think about the condition in my family, I get upset. | 1 | 2 | 3 | 4 | 5 | 6 | 7 |
| 5 | I don’t know where to go to get the medical help I / my family need(s). | 1 | 2 | 3 | 4 | 5 | 6 | 7 |
| 6 | I can see that good things have come from having this condition in my family. | 1 | 2 | 3 | 4 | 5 | 6 | 7 |
| 7 | I can control how this condition affects my family. | 1 | 2 | 3 | 4 | 5 | 6 | 7 |
| 8 | I feel positive about the future. | 1 | 2 | 3 | 4 | 5 | 6 | 7 |
| 9 | I am able to cope with having this condition in my family. | 1 | 2 | 3 | 4 | 5 | 6 | 7 |
| 10 | I don’t know what could be gained from each of the options available to me. | 1 | 2 | 3 | 4 | 5 | 6 | 7 |
| 11 | Having this condition in my family makes me feel anxious. | 1 | 2 | 3 | 4 | 5 | 6 | 7 |
| 12 | I don’t know if this condition could affect my other relatives (brothers, sisters, aunts, uncles, cousins). | 1 | 2 | 3 | 4 | 5 | 6 | 7 |
| 13 | In relation to the condition in my family, nothing I decide will change the future for my children / any children I might have. | 1 | 2 | 3 | 4 | 5 | 6 | 7 |
| 14 | I understand the reasons why my doctor referred me to the clinical genetics service. | 1 | 2 | 3 | 4 | 5 | 6 | 7 |
| 15 | I know how to get the non-medical help I / my family needs (e.g. educational, financial, social support). | 1 | 2 | 3 | 4 | 5 | 6 | 7 |
| 16 | I can explain what the condition means to people outside my family who may need to know (e.g. teachers, social workers). | 1 | 2 | 3 | 4 | 5 | 6 | 7 |
| 17 | I don’t know what I can do to change how this condition affects me / my children. | 1 | 2 | 3 | 4 | 5 | 6 | 7 |
| 18 | I don’t know who else in my family might be at risk for this condition. | 1 | 2 | 3 | 4 | 5 | 6 | 7 |
| 19 | I am hopeful that my children can look forward to a rewarding family life. | 1 | 2 | 3 | 4 | 5 | 6 | 7 |
| 20 | I am able to make plans for the future. | 1 | 2 | 3 | 4 | 5 | 6 | 7 |
| 21 | I feel guilty because I (might have) passed this condition on to my children. | 1 | 2 | 3 | 4 | 5 | 6 | 7 |
| 22 | I am powerless to do anything about this condition in my family. | 1 | 2 | 3 | 4 | 5 | 6 | 7 |
| 23 | I understand what concerns brought me to the clinical genetics service. | 1 | 2 | 3 | 4 | 5 | 6 | 7 |
| 24 | I can make decisions about the condition that may change my child(ren)’s future / the future of any child(ren) I may have. | 1 | 2 | 3 | 4 | 5 | 6 | 7 |

McAllister M, Wood A, Dunn G, Shiloh S, Todd C. (2011) The Genetic Counseling Outcome Scale: a new patient-reported outcome measure for clinical genetics services. *Clinical Genetics*, 79, 413–424.
