# Supplementary material for: Using Patient-Reported Outcome Measures for Quality Improvement in Clinical Genetics: an Exploratory Study
Source: J Genet Couns. 2017 Mar 9;26(5):1017–28. doi: 10.1007/s10897-017-0079-6 (PMC5582073; doi:10.1007/s10897-017-0079-6)
Supplement: Supplementary file 3 — (DOCX 44 kb) [file 10897_2017_79_MOESM3_ESM.docx]

Supplement C: Skirton Audit Tool

Using the scale below, circle a number next to each statement to indicate how much you agree with the statement. Please answer all the questions. For questions that are not applicable to you, please choose option 4 (neither agree nor disagree).

agree

slightly disagree

neither agree nor disagree

slightly agree

disagree

strongly agree

strongly disagree

1 = strongly disagree

2 = disagree

3 = slightly disagree

4 = neither disagree nor agree

5 = slightly agree

6 = agree

7 = strongly agree

| 1 | I have more understanding of how the condition affects the person who has it | 1 | 2 | 3 | 4 | 5 | 6 | 7 |
| --- | --- | --- | --- | --- | --- | --- | --- | --- |
| 2 | I have more understanding of how the condition develops during a person's lifetime.  need to know. | 1 | 2 | 3 | 4 | 5 | 6 | 7 |
| 3 | I have more understanding of what causes the condition. | 1 | 2 | 3 | 4 | 5 | 6 | 7 |
| 4 | I understand the risk of my relatives getting the condition. | 1 | 2 | 3 | 4 | 5 | 6 | 7 |
| 5 | I have more understanding of the severity of the condition. | 1 | 2 | 3 | 4 | 5 | 6 | 7 |
| 6 | I feel more positive. | 1 | 2 | 3 | 4 | 5 | 6 | 7 |
| 7 | I have greater peace of mind. | 1 | 2 | 3 | 4 | 5 | 6 | 7 |
| 8 | I feel more negative. | 1 | 2 | 3 | 4 | 5 | 6 | 7 |
| 9 | I feel I can adapt better to changes. | 1 | 2 | 3 | 4 | 5 | 6 | 7 |
| 10 | I am more able to ask for help if I need it. | 1 | 2 | 3 | 4 | 5 | 6 | 7 |
| 11 | I feel more in tune with others close to me.  . | 1 | 2 | 3 | 4 | 5 | 6 | 7 |
| 12 | My main questions were answered.  sisters, aunts, uncles, cousins). | 1 | 2 | 3 | 4 | 5 | 6 | 7 |
| 13 | I did not feel comfortable.  the future for my children / any children I might have. | 1 | 2 | 3 | 4 | 5 | 6 | 7 |
| 14 | I could not understand what I was told.  service. | 1 | 2 | 3 | 4 | 5 | 6 | 7 |
| 15 | I felt I was being told what to do.  educational, financial, social support). | 1 | 2 | 3 | 4 | 5 | 6 | 7 |
| 16 | I felt treated as an individual.  may need to know (e.g. teachers, social workers). | 1 | 2 | 3 | 4 | 5 | 6 | 7 |
| 17 | I wish I had not been referred to the genetics service.  my children. | 1 | 2 | 3 | 4 | 5 | 6 | 7 |
| 18 | I could not understand the explanations I was given. | 1 | 2 | 3 | 4 | 5 | 6 | 7 |
| 24 | I can make decisions about the condition that may change my child(ren)’s future / the future of any child(ren) I may have. | 1 | 2 | 3 | 4 | 5 | 6 | 7 |

Skirton H, Parsons E, Ewings P. (2005) Development of an audit tool for genetics services. *Am J Med Genet*, 132A, 122-127.
