# Supplementary material for: Using Patient-Reported Outcome Measures for Quality Improvement in Clinical Genetics: an Exploratory Study
Source: J Genet Couns. 2017 Mar 9;26(5):1017–28. doi: 10.1007/s10897-017-0079-6 (PMC5582073; doi:10.1007/s10897-017-0079-6)
Supplement: Supplementary file 4 — (DOCX 17 kb) [file 10897_2017_79_MOESM4_ESM.docx]

**Supplementary D: Interview Guide for ‘Exploring the views of genetics health professionals on use of patient-reported outcome data for quality improvement in clinical genetics services’.**

This guide will only act as a reference to prompt discussion and focus topics throughout the interview. It is meant to act as an outline and is meant to be flexible.

**Introduction**

- Explanation of research and answering any questions or concerns
- Outline of the interview format
- Reminder that participant can stop the interview at any time
- Explanation that notes may be taken and that participant may be asked to clarify ideas
- Review of consent form
- Assurance that confidentiality and anonymity will be maintained

**Background**

- Tell me about yourself e.g. type of clinician (genetic counselor, clinical geneticist), years of clinical experience, how long worked at All Wales Medical Genetics Service (AWMGS), any specific interest or experience in service quality improvement etc.)
- Could you tell me a bit about how you became involved in the initiative to use patient questionnaire data for service quality improvement in the All Wales Medical Genetics Service (AWMGS)?

**Acceptability and usefulness of the questionnaires:**

What do you think of the questionnaires that are being used in this initiative?

- Genetic Counseling Outcome Scale
- EQ-5D
- AWMGS satisfaction questionnaire

What are your views about usefulness of the data collected on the questionnaires?

- Genetic Counseling Outcome Scale
- EQ-5D
- AWMGS satisfaction questionnaire

How do you think the information collected on these questionnaires could be used by the clinical service to improve patient care?

**Resources required:**

How easy or hard do you think it is for a clinical service to collect these data?

What challenges does collecting the data pose to the clinical team, including administrative staff?

Thinking about how these data might be used, how easy or hard do you think it is for a clinical service to use these data to improve the service provided?

What challenges does reviewing the data pose to the clinical team?

What challenges does using the data for service quality improvement pose to the clinical team?

**Barriers and enablers to implementation:**

What strengths do administrative staff have that can help make this kind of initiative successful?

What training and support needs do administrative staff have in order to make this kind of initiative successful?

What strengths do clinical staff have that can help make this kind of initiative successful?

What training and support needs do clinical staff have in order to make this kind of initiative successful?

**Methods for interpreting and using the PRO data for continuous quality improvement in patient care:**

What approaches, if any, has your team tried, up to now, to understand the questionnaire data?

What approaches, if any, has your team tried, up to now, to use the questionnaire data?

What approaches, if any, has your team tried, up to now, to think about the implications of these data for the quality of the service provided by the AWMGS?

What approaches, if any, has your team tried, up to now, to make changes to the service provided by the AWMGS, based on the questionnaire feedback?

**Spreadability beyond AWMGS**

In your view, what is the feasibility of moving this approach beyond the AWMGS (to other clinical genetics services e.g. in England)?

In your view, what are the challenges of moving this approach beyond the AWMGS (to other clinical genetics services e.g. in England)?

**Specifics of how the initiative is working in AWMGS**

To make this initiative work, how often do you think the team needs to meet to discuss the data collected and how the data might best be used?

To make this initiative work, what do you think is the best way to manage the meetings?

To make this initiative work, what do you think is the best way to record any decisions made, actions arising etc.?

To make this initiative work, what do you think is the best way to take things forward from here?

What do you think is the best way to determine whether the initiative is working well?

**Closing**

- Is there anything additional we have not touched on that you would like to discuss about your experience?
- Thank you for your time
